# Harvest Festivals

**Published:** 1891-10-31

**Authors:** 


### Passing Topics.

### HARVEST FESTIVALS.

The harvest festival is an established institution of the country and a recognised and popular vehicle for charitable endeavours. Wherever there is a church, or for that matter, wherever there is a barn, or room, that does duty for a church, there it is the custom to hold high festival upon some chosen day in this period of mid-autumn. The clergy and the laity are alike concerned in the ceremonial, and prodigious are the efforts made to afford material evidence of the keen interest taken in the time-honoured custom which is a survival and a reflection of the doings of those primitive days when an actual tithe of the earth's yield was offered up in sacrifice to Heaven.

There must be few people who are altogether insensible of the beauty of the sentiment pourtrayed in a harvest thanksgiving service, provided always that it embodies a genuine and devout feeling of gratitude to the Giver of all good for blessings vouchsafed. But precisely in the ratio that we see room to doubt the reality of the praise and thanksgiving, do we question the propriety of the mere ceremonial. In this present disastrous year of storm and deluge, when loss and even ruin threatens many a husbandman, and when it is not too much to say that positive famine would impend, were we, as a people, dependent upon the unaided yield of our own farms, it is difficult to understand why it has not occurred to the organisers of festivals in certain sorely-stricken districts that it might be well to introduce some modifications into proceedings which are appropriate enough in years of plenty, but under such adverse conditions as we have suffered appear calculated to provoke the scornful comments of the sceptic.

In many agricultural parishes the absence of seasonable weather has culminated in a veritable catastrophe, yet we have scarcely heard of a case where a church has voluntarily surrendered a custom which in circumstances so untoward would certainly appear to be better honoured in the breach than in the observance. Of course, we do not need to be reminded that occasion for thankfulness never fails us. But in the Harvest Festival Service we are invited to come together not to offer a general thanksgiving for life's every day mercies, but in order to render glory to the Deity for a specific gift and blessing. And if in His wisdom that blessing is denied to a particular parish, is it reasonable or is it reverent to offer empty and necessarily inappropriate phrases? If this question be not seriously asked and answered in the way common sense appears to require, we fear that the beautiful custom must languish and die out for the reason that it will have become meaningless.

Three weeks ago, in such storm of wind and rain as is uncommon even upon the rough hillsides which lie open to the Atlantic gales, the shrill bell of a little village church summoned worshippers to the Harvest Festival. In the adjacent fields, well open to view from the interior of the church itself, so long as the waning autumn light sufficed long lines of sheaves stood black and dripping in the pitiless downpour. In the hollow, the brook was running bank-high; beyond, were waves of corn lying as it had fallen before the sickle while the now full moon was young, while round about were acres of turnips and mangolds stunted for want of warmth, and potatoes rotting in the sodden soil. Could it be supposed that this was an occasion for exuberant thankfulness? The parson might have counselled patience but surely not rejoicing. What the village thought was shown in the meagre gathering, which consisted of the vicar's family, half-a-dozen women, the village constable, whom we strongly suspect to have been a martyr to his sense of duty, and one or two stray visitors. The collection was for the County Hospital, and we much fear that its proportions only too truthfully displayed the feeling of the parish. Agricola.

